# Codman maneuver for biceps tendonitis: a prospective evaluation of a forgotten examination maneuver

**DOI:** 10.1016/j.jseint.2024.12.011

**Published:** 2025-01-16

**Authors:** John M. Kopriva, Alexander M. Dawes, Hayden L. Cooke, Julianne W. Gillis, Michael B. Gottschalk, Eric R. Wagner

**Affiliations:** Department of Orthopaedic Surgery, Emory University School of Medicine, Atlanta, GA, USA

**Keywords:** Rotator cuff tendinopathy, Biceps tendinitis, Codman maneuver, VAS pain, Biceps corticosteroid injections, Anterior shoulder pain

## Abstract

**Background:**

Many physical exam maneuvers for shoulder pain exist, but the sensitivity and specificity are repeatedly found to be subpar, even in the most utilized maneuvers. Rotator cuff tendinopathy and biceps tendonitis are common pain generators that often go undifferentiated. The purpose of this study was to examine a century-old examination maneuver to help identify biceps tendinitis in patients presenting with anterior shoulder pain.

**Methods:**

Twenty-seven consecutive patients who presented to clinic in a 10-month period with shoulder pain, no osseous concerns on radiographs, and subsequently received an ultrasound-guided corticosteroid injection (CSI) at the bicipital groove were prospectively enrolled. Our updated Codman maneuver was performed along with full and empty can tests in multiple planes. Visual analog scale (VAS) pain scores for each maneuver and isometric strength for the full and empty can tests were compared between patients who improved with ultrasound-guided CSI at the bicipital groove and those who did not.

**Results:**

24 of 27 patients responded favorably to CSI. Of these 24 patients, mean VAS scores across full and empty can maneuvers ranged from 4.4 (±3.2) to 5.5 (±3.0). Codman for biceps had a significantly higher mean VAS at 8.8 (±1.0). Conversely, Codman for supraspinatus had a significantly lower mean VAS score of 3.2 (±2.8).

**Conclusion:**

The described Codman maneuver for biceps was correlated with patients who benefited from an ultrasound-guided CSI at the bicipital groove. Combining the Codman maneuver with patient-reported VAS pain scale is a promising maneuver for biceps tendinopathy.

The physical exam remains a key component of the diagnostic algorithm for shoulder pain. However, few maneuvers have been shown to reliably isolate specific diagnoses.^5^^,^^7^^,^^10^^,^^16^^,^^21^ For example, two of the most common shoulder pathologies that present with pain in the United States are biceps tendonitis (long head) and rotator cuff tendinopathy.^18^ With the supraspinatus and subscapularis insertions flanking the bicipital groove, symptoms and provocative tests often overlap. Common special tests for biceps tendonitis include Speed’s test^14^ and Yergason’s test.^9^ Typical maneuvers for rotator cuff tendinopathy include subacromial painful arc test,^22^ the “empty can” Jobe test,^11^ and “full can” test for rotator cuff tears.^12^ Current literature has yet to document superior sensitivity and specificity for any one of these tests.^3^^,^^7^^,^^8^

Almost a century ago, in one of the first orthopedic textbooks (Fig. 1), E.A. Codman described his method of palpating a supraspinatus tear based on reliable anatomic landmarks.^2^ In 1934, without magnetic resonance imaging (MRI), Dr. Codman indicated patients for surgery by palpating what he called the “sulcus” in reference to the defect in the torn supraspinatus. This spot, just off the anterolateral rim of the acromion, would be painful both to direct palpation and with passive elevation of the arm as that sulcus engaged the subacromial space. He also noted the ability to easily find the nearby bicipital groove with the long head tendon. Unfortunately, Codman’s maneuver has not been well-preserved or further developed through the subsequent years of orthopedic teaching. Concomitantly, no single provocative test has been shown to discriminate between supraspinatus tears and biceps tendinopathies accurately and reliably.^3^^,^^13^

**Figure 1:**
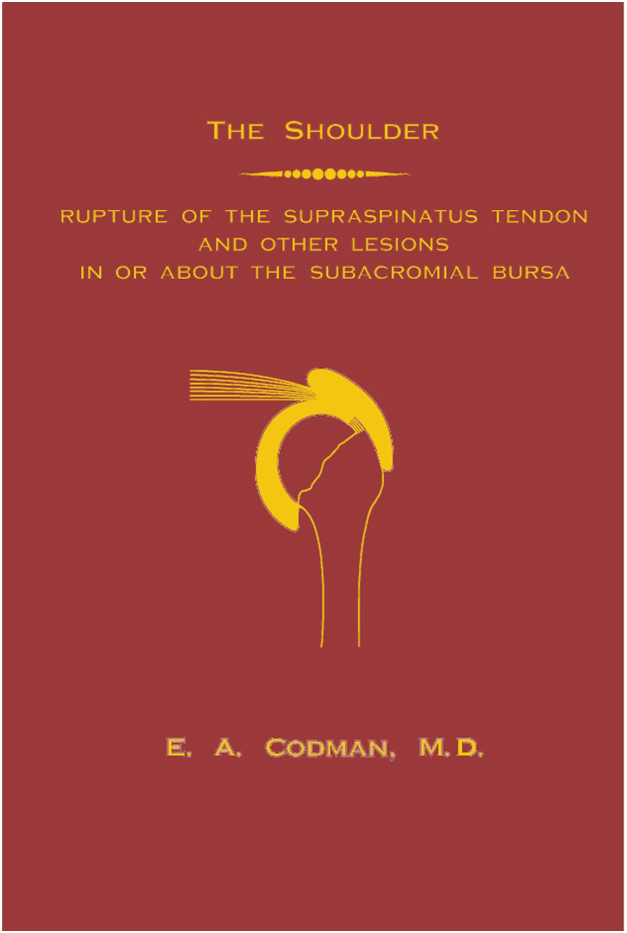
Picture of E.A. Codman’s “The Shoulder, Rupture of the Supraspinatus Tendon and Other Lesions in or about the Subacromial Bursa”. (Reprinted with permission from The Shoulder, Rupture of the Supraspinatus Tendon and Other Lesions in or about the Subacromial Bursa. Boston, MA: Thomas Todd Company; 1934.)

We believe Dr. Codman’s understanding of shoulder anatomy and patient positioning during the physical exam is underutilized as a maneuver to differentiate pain in the shoulder. Furthermore, relying on advanced imaging after a nondiagnostic exam can lead to incidental asymptomatic findings, especially in the shoulder.^4^ The purpose of this study was to describe our updated Codman maneuver and to evaluate its effectiveness identifying patients who benefit from ultrasound-guided corticosteroid injections (CSI) at the bicipital groove (diagnostic for biceps tendonitis). We hypothesize that the Codman maneuver better identifies biceps tendonitis than traditional “full can” and “empty can” provocative maneuvers.

## Materials and methods

After institutional review board approval, all patients presenting to the clinics of two shoulder subspecialists at a single institution were evaluated for this prospective study from March 2019 to December 2019. Patients were included if they were 18 years of age or older, presented with isolated anterior shoulder pain of unknown etiology, and elected to receive a subsequent ultrasound-guided CSI at the bicipital groove. Patients were excluded if they were diagnosed with severe glenohumeral arthritis on radiograph, had a history of inflammatory arthritis, prior biceps tenodesis or tenotomy, had multiple areas of disabling pain around the shoulder, or found to have a fracture or tumor of the upper extremity.

Patients were recommended CSI based on their history and physical examination. Both fellowship-trained examiners were blinded to previous imaging and clinical notes. Empty and full can test maneuvers as well as the Codman maneuver were standardized before data collection began. Patients recorded visual analog scale (VAS) pain scores for each maneuver. The CSI was administered at the bicipital groove by a physician with board certification in ultrasound.

Both prospective and retrospective chart review were conducted. The patients’ names, medical record numbers, age at time of evaluation, gender, body mass index, presence of diabetes, smoking status, duration of pain, and physical exam findings at the time of the clinic visit were recorded. Responses to injections were recorded at the 2-week time point after the injection. Biceps tendinitis was confirmed if the patient had a positive response to an ultrasound-guided injection of the bicipital groove.^20^ The positive response was defined as a VAS pain score of 2 or lower after the injection or an improvement of >5 points from pre-to postinjection.

### Statistical modeling

This study was powered using VAS pain with a mean of 1 VAS point difference. Using a power of 0.90, it was determined that 28 participants should be enrolled. Data were stored on an excel spreadsheet in a password-protected, Health Insurance Portability and Accountability Act (HIPPA)-compliant data bank. Each variable was used as a nominal variable when possible (eg, smoking status) or a continuous variable if needed (eg, pain on a scale of 1-10). We performed statistical analysis using Mann–Whitney U tests to compare means for non-normally distributed data. Statistical significance was interpreted as *P* < .05. JMP Pro Software (SAS Institute Inc., Cary, NC, USA) and SPSS Statistics (version 29.0.1; IBM, Armonk, NY, USA) were used for analysis.

## Codman maneuver

**Figure 2:**
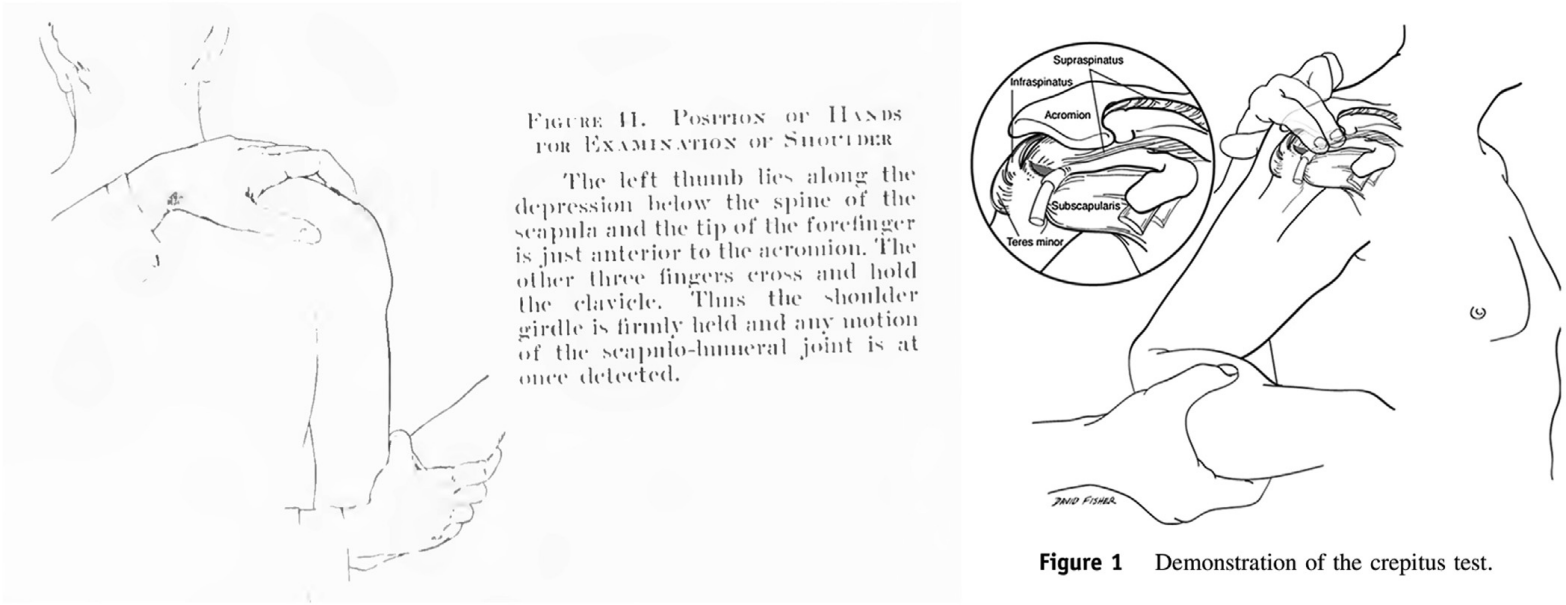
Demonstrations of prior “Codman maneuvers” from E.A. Codman’s “The Shoulder” (*left*) and Ponce et al’s “Rotator cuff crepitus: could Codman really feel a cuff tear”? (*right*). (Reprinted from J Shoulder Elbow Surg 2014;23(7):1017-22; Ponce BA, Kundukulam JA, Sheppard ED, Determann JR, McGwin G, Narducci CA, et al Rotator cuff crepitus: could Codman really feel a cuff tear, with permission from Elsevier.)

**Figure 3:**
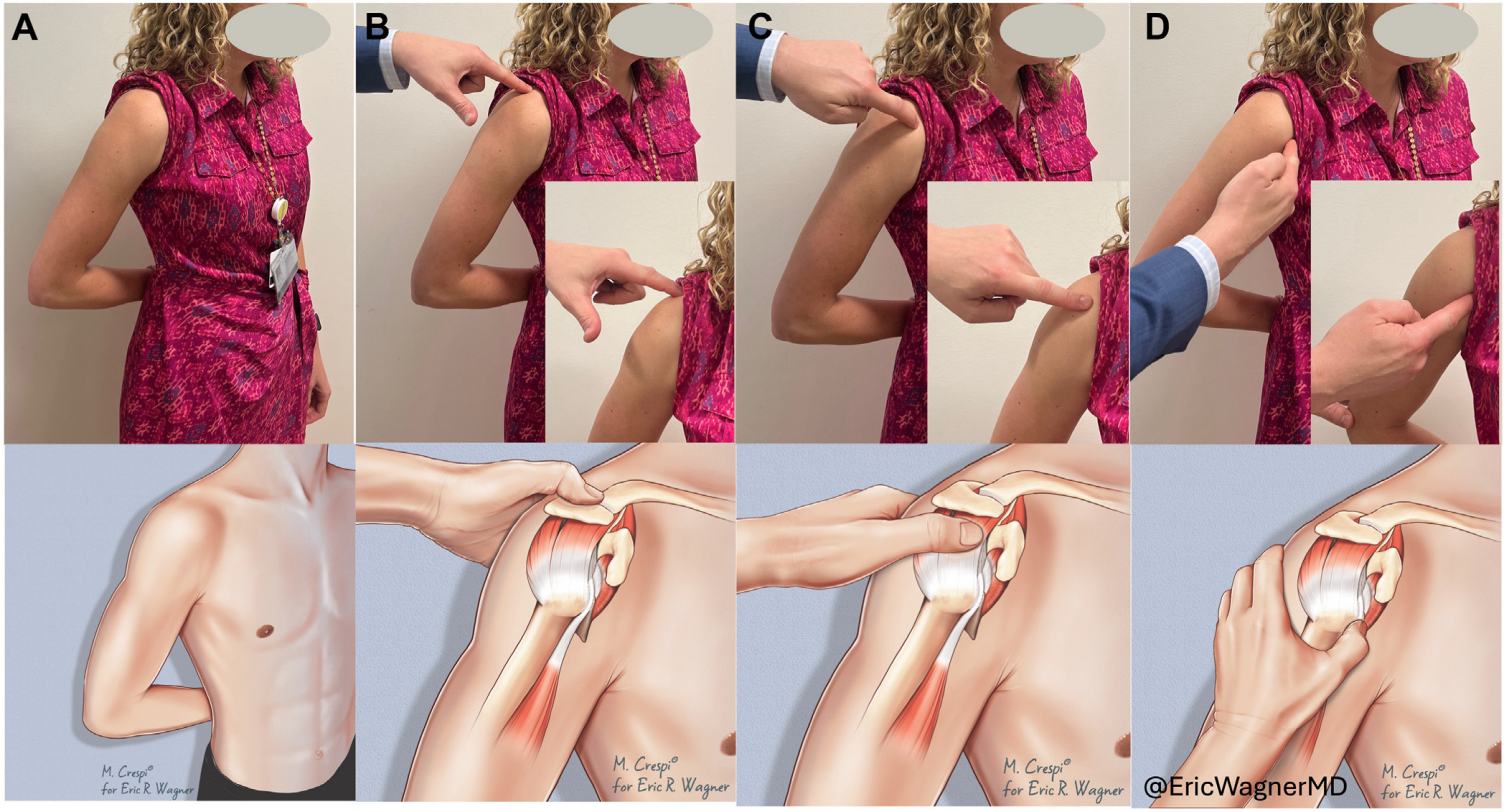
The Codman maneuver. (**A**) Positioning of the patient’s hand at the posterior aspect of the iliac wing. Examiner palpating the (**B**) acromion, (**C**) supraspinatus insertion, and (**D**) the long head of biceps.

**Figure 4:**
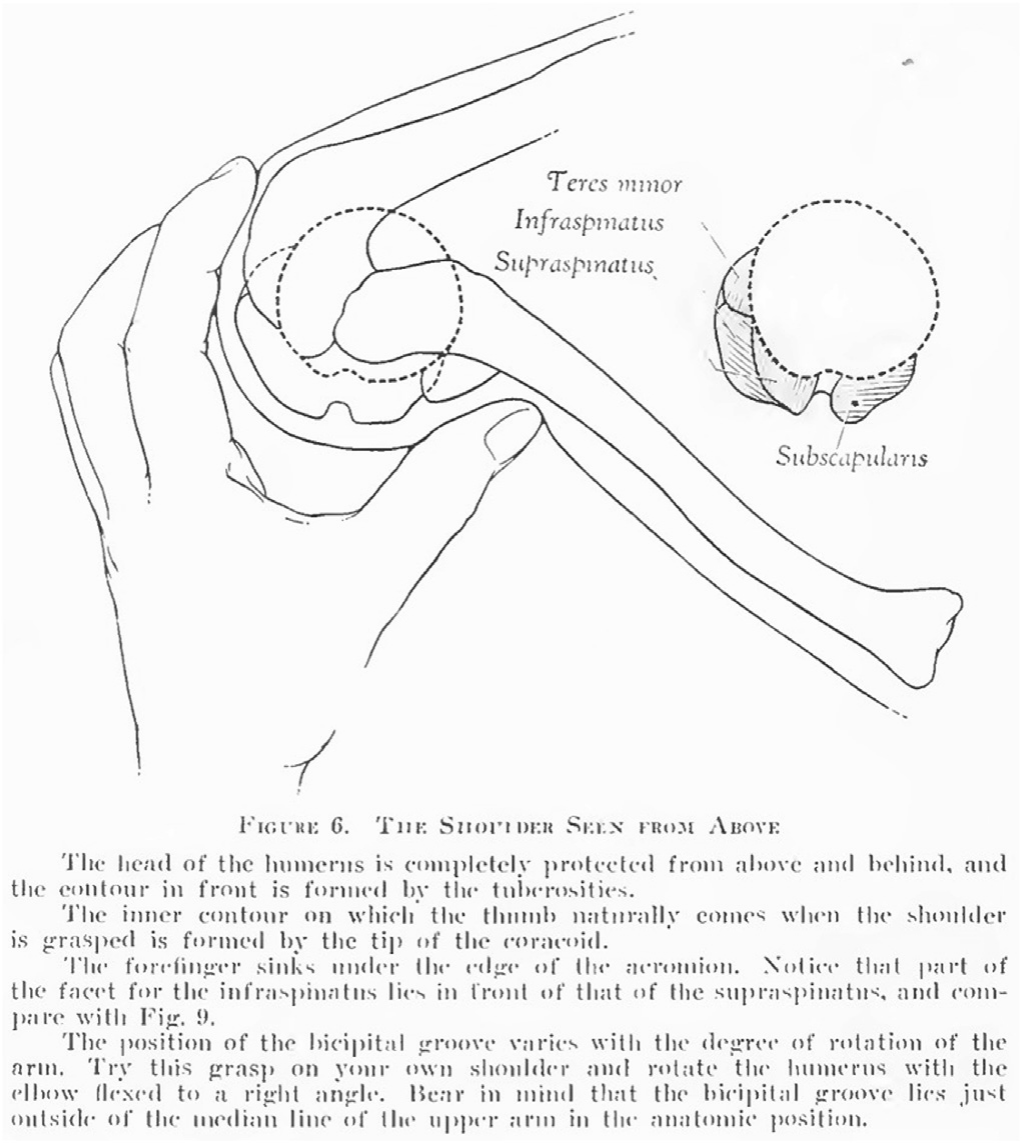
Demonstration of the internal rotation and extension of the humerus during the Codman maneuver, bringing the bicipital groove toward the midline and exposing more of the supraspinatus footprint on the greater tuberosity. Figure from E.A. Codman’s “The Shoulder”.

Codman originally described his exam with the humerus in a neutral position—patient with the arm adducted at the side, elbow flexed to 90°, and forearm directly in line with the sagittal plane (Fig. 2).^2^ We have modified this positioning to better deliver the bicipital groove and supraspinatus footprint. The patient is asked to relax the arm while the examiner takes the patient’s hand and places it on their posterior belt line, resting the hand on the posterior aspect of the ipsilateral iliac crest (Fig. 3). This positioning internally rotates and extends the humerus, bringing the bicipital groove toward the midline and exposing more of the supraspinatus footprint on the greater tuberosity (Fig. 4). The examiner then presses firmly on the site of insertion of the supraspinatus, which is now approximately 1 cm inferior to the anterolateral border of the acromion (Fig. 3). The examiner then palpates the long head of biceps tendon as it courses through the bicipital groove, 2-3 cm inferior to the anterior edge of the acromion (Fig. 3). At each location, the patient is asked to rate their VAS pain on a scale of 0-10.^6^

### Traditional shoulder maneuvers

**Figure 5:**
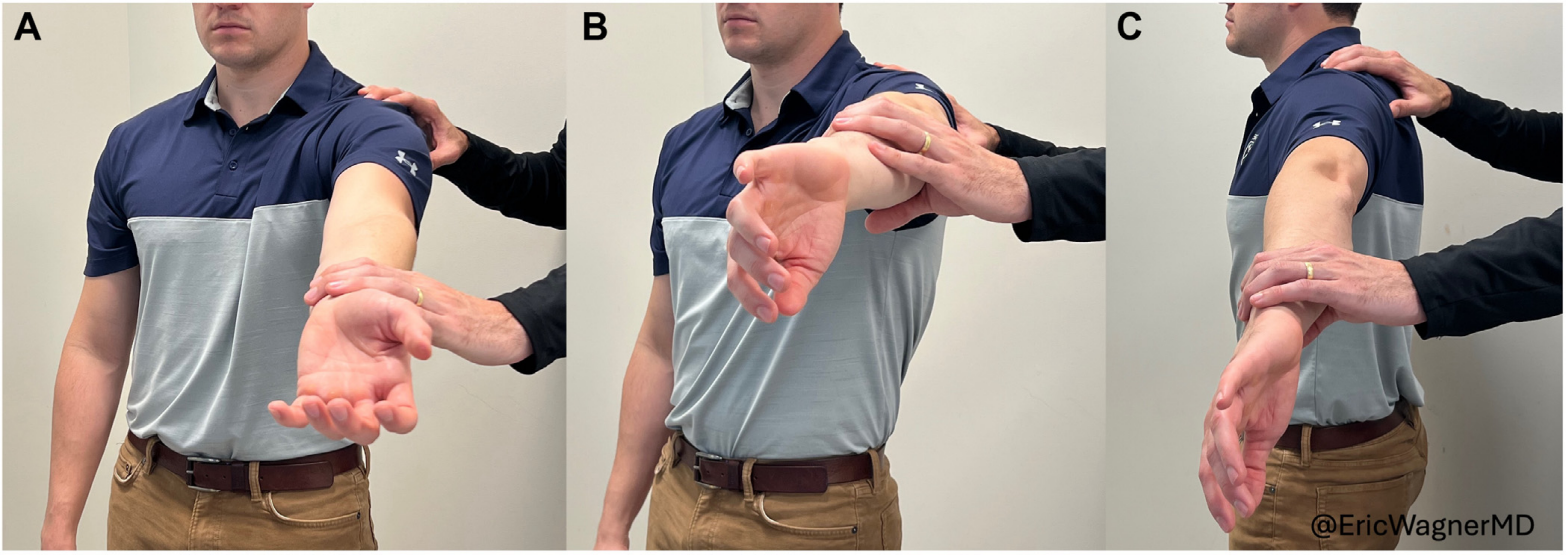
Example of full and empty can maneuver in three planes (**A**) forward (full), (**B**) scapular (empty), and (**C**) abduction (empty). (Reprinted with permission from “The Shoulder, Rupture of the Supraspinatus Tendon and Other Lesions in or about the Subacromial Bursa”. Boston, MA: Thomas Todd Company; 1934.)

Jobe’s, Speed’s, and O’brien’s tests are each traditional shoulder exam maneuvers. Each test involves straight arm elevation in the forward, scapular, or coronal plane with the arm supinated or pronated. For completeness and reproducibility, we elected to evaluate all combinations of “full” and “empty can” tests in the three planes (Fig. 5). Empty can in the scapular plane represents the “Jobe’s test”^11^ for supraspinatus pathology. Full can in forward flexion represents the “Speed’s test”^17^ for biceps tendon pathology. Empty and full can tests comparison in forward flexion represents the “O’Brien test”^15^ for superior labrum and biceps pathology. The patient’s VAS pain scores were recorded for each of these positions with each test. Additionally, isometric strength measurements in each of the six full and empty can position were obtained using a handheld dynamometer.

## Results

Forty-seven patients who presented with isolated anterior shoulder pain were identified for study eligibility, and 27 ultimately agreed to undergo ultrasound-guided CSI into the bicipital groove. The others declined ultrasound-guided injections and thus were not included in the study. Of the 27 included patients, the majority were women (67%). The mean age at the time of initial evaluation was 62.6 years (range, 32-77 years). The mean duration of symptoms was 12 months (range, 1-60 months). The mean body mass index was 31.6 kg/m^2^ (range, 21-48), 5 (19%) patients had diabetes mellitus, and 4 (15%) had been diagnosed with either depression or anxiety (Table I).

**Table I:** Patient demographics.

Variable	N = 27
Age	62.6 (32-77)
Sex	
Male	9 (33%)
Female	18 (67%)
BMI	31.6 (21-48)
Diabetes	5 (19%)
Anxiety or depression	4 (15%)
Symptom duration	11.5 (1-60)
Presented as average (range) or number (percent)	

*BMI*, body mass index.

Across all tests, including the Codman maneuver, the mean VAS scores were higher in the painful shoulder than the unaffected contralateral shoulder (Table II). Similarly, isometric strength tests for all full and empty can tests in three planes were decreased in the painful shoulder compared to the unaffected contralateral shoulder (Table II).

**Table II:** Patient baseline strength and pain VAS for unknown etiology.

		Affected shoulder	Unaffected shoulder	*P* value
Empty can				
Forward flexion	Pain (VAS)	4.4 (±3.2)	0.9 (±1.8)	**<.001**
	*Strength (kg)*	*7.8 (±4.0)*	*9.8 (±3.6)*	.065
Elevation (scapular plane)	Pain (VAS)	4.8 (±3.1)	0.6 (±1.0)	**<.001**
	*Strength (kg)*	*7.2 (±4.4)*	*9.7 (±3.2)*	**.028**
Abduction	Pain (VAS)	5.5 (±2.9)	0.8 (±1.5)	**<.001**
	*Strength (kg)*	*6.7 (±3.4)*	*9.2 (±3.0)*	**.013**
Full can				
Forward flexion	Pain (VAS)	4.9 (±2.4)	0.4 (±0.9)	**<.001**
	*Strength (kg)*	*7.3 (±3.7)*	*9.2 (±3.0)*	.059
Elevation (scapular plane)	Pain (VAS)	4.6 (±2.6)	0.6 (±1.3)	**<.001**
	*Strength (kg)*	*7.7 (±3.4)*	*9.5 (±3.1)*	**.036**
Abduction	Pain (VAS)	5.3 (±2.8)	0.8 (±1.5)	**<.001**
	*Strength (kg)*	*7.0 (±4.1)*	*9.3 (±3.2)*	**.01**
Codman				
Supraspinatus	Pain (VAS)	3.2 (±2.8)	0.7 (±0.9)	**<.001**
Biceps	Pain (VAS)	8.8 (±1.0)	1.2 (±1.3)	**<.001**

*VAS*, Visual analog scale.

Presented as mean (±standard deviation) for each category.

Bold text denotes statistical significance.

24 out of 27 patients (89%) found significant relief from the ultrasound-guided CSI into the bicipital groove. When comparing patients with successful CSI to those who did not find relief (Table III), the mean VAS pain scores for all traditional tests were no different. Full and empty can testing in all three planes had mean VAS pain scores from 4.4 (±3.2) to 5.5 (±3.0). In contrast, the mean VAS pain score was elevated using Codman for biceps in the group that responded to CSI at 8.8 (±1.0) (*P* ≤ .001). This mean VAS of 8.8 (±1.0) was significantly higher than any other test in those who responded to CSI (Table IV). Codman for supraspinatus had the lowest mean VAS pain score in the group that responded to CSI at 3.2 (±2.8), nearly significantly lower than nonresponders with a mean VAS of 6.3 (±1.5) (*P* = .7). There was no difference in the mean isometric strengths between the responder and nonresponder groups in all planes of full and empty can tests.

**Table III:** Patient baseline strength and pain VAS for patients with or without biceps tendinitis.

		Response to CSI (N = 24)	No response (N = 3)	*P* value
Empty can				
Forward flexion	Pain (VAS)	4.4 (±3.2)	3.7 (±2.1)	.689
	*Strength (kg)*	*7.8 (±4.0)*	*8.3 (±3.7)*	.689
Elevation (scapular plane)	Pain (VAS)	4.8 (±3.1)	4.3 (±3.2)	.743
	*Strength (kg)*	*7.2 (±4.4)*	*7.7 (±3.1)*	.856
Abduction	Pain (VAS)	5.5 (±3.0)	4.0 (±2.6)	.437
	*Strength (kg)*	*6.7 (±3.4)*	*6.3 (±2.3)*	.799
Full can				
Forward flexion	Pain (VAS)	4.9 (±2.4)	4.0 (±2.6)	.437
	*Strength (kg)*	*7.3 (±3.7)*	*7.8 (±3.4)*	.689
Elevation (scapular plane)	Pain (VAS)	4.6 (±2.6)	4.0 (±2.6)	.689
	*Strength (kg)*	*7.7 (±3.4)*	*6.7 (±4.9)*	.914
Abduction	Pain (VAS)	5.3 (±2.8)	4.3 (±2.5)	.583
	*Strength (kg)*	*7.0 (±4.1)*	*6.3 (±4.7)*	.971
Codman				
Supraspinatus	Pain (VAS)	3.2 (±2.8)	6.3 (±1.5)	.07
Biceps	Pain (VAS)	8.8 (±1.0)	2.7 (±2.1)	**<.001**

*VAS*, Visual analog scale; *CSI*, Corticosteroid injection.

Presented as mean (±standard deviation) for each category.

Bold text denotes statistical significance.

**Table IV:** VAS pain scores for each test compared to Codman for biceps in +Biceps group.

+Biceps	VAS pain scores	Codman biceps VAS	*P* value
Empty can			
Forward flexion	4.4 (±3.2)	8.8 (±1.0)	**<.001**
Elevation (scapular plane)	4.8 (±3.1)	8.8 (±1.0)	**<.001**
Abduction	5.5 (±3.0)	8.8 (±1.0)	**<.001**
Full can			
Forward Flexion	4.9 (±2.4)	8.8 (±1.0)	**<.001**
Elevation (scapular plane)	4.6 (±2.6)	8.8 (±1.0)	**<.001**
Abduction	5.3 (±2.8)	8.8 (±1.0)	**<.001**
Codman			
Supraspinatus	3.2 (±2.8)	8.8 (±1.0)	**<.001**

*VAS*, Visual analog scale; *+Biceps*, Patients with a response to biceps tendon corticosteroid injection.

Presented as mean (±standard deviation) for each category.

Bold text denotes statistical significance using a Mann-Whitney U test for each comparison.

## Discussion

For the evaluation of a painful shoulder, a plethora of shoulder exam maneuvers have been described. However, typical shoulder tests are consistently fraught with only moderate sensitivity and specificity.^1^^,^^3^^,^^7^, ^8^, ^9^ Furthermore, MRI is known to show asymptomatic pathology,^4^ making it difficult to identify the current pain generator. Almost a century ago, E.A. Codman described his method of reliably palpating the supraspinatus insertion and biceps tendon,^2^ but his maneuver has not been well-preserved in orthopedic teaching. The purpose of this investigation was twofold—1) to describe our updated Codman maneuver for biceps and supraspinatus pain and 2) to compare its effectiveness in diagnosing biceps tendonitis to traditional tests.

We prospectively compared the Codman manuever to traditional “empty can” and “full can” tests. Using response to an ultrasound-guided CSI in the bicipital groove as a diagnostic tool for biceps tendonitis, preinjection VAS pain scores were compared between maneuvers in those with positive responses to those who did not improve with CSI. Codman for biceps best identified patients who responded to CSI with the highest average VAS score of 8.8, which was significantly greater than nonresponders (*P* < .001). Traditional empty and full can tests did not differentiate responders to CSI, with mean VAS scores between 4.4 and 5.5, all significantly lower than Codman for biceps (*P* < .001). Codman for supraspinatus had the lowest mean VAS score in the responder group at 3.2—a stark difference from the Codman for biceps at 8.8 (*P* < .001).

The Codman maneuver used in this study is an evolution of E.A. Codman’s originally described palpation maneuver. Wolf and Agrawal described their adaptation of the Codman maneuver as “transdeltoid palpation (the rent test)” to identify rotator cuff tears.^23^ Compared to Codman’s neutral positioning of the arm, their adaptation describes the examiner holding the patient’s relaxed arm by the flexed elbow and passively extending the humerus. From this extended position, they describe internally and externally rotating the humerus to allow for palpation of the cuff footprint and possible tear. Similarly, Ponce et al evaluated “rotator cuff crepitus” as an exam finding in rotator cuff tears,^19^ referencing Codman (Fig. 2). They, too, note the use of passive extension with internal and external rotation of the arm to identify anatomic landmarks and rotator cuff tears. We recommend a static position for the exam placing the patient’s hand over the posterior iliac wing (Fig. 3), which internally rotates and extends the humerus, delivering the greater tuberosity and bicipital groove (Fig. 4). From this position, the supraspinatus tendon will be about 1 cm inferior to the anterolateral edge of the acromion and the biceps tendon 2-3 cm inferior to the anterior edge of the acromion (Fig. 3).

In contrast to the full and empty can tests, the Codman maneuver is a passive exam for the patient. As pointed out by Ponce et al, this critical difference from other provocative tests eliminates patient effort as a confounding factor.^19^ Perhaps most apparent in the isometric strength measurements, pain can limit effort or ability. Strength measurements were globally decreased in the painful arm compared to the contralateral control (Table II). Furthermore, when comparing responders to nonresponders, isometric strength for empty and full can maneuvers did not differentiate patients. In contrast, the Codman maneuver reduces pain as a confounding factor by removing patient participation and maintaining a static position. The arm extension and internal rotation places the anterior greater tuberosity in a position to be easily palpated and places a passive stretch on the supraspinatus and biceps tendons. Thus, if they are pathologically injured, pain with palpation theoretically should be elicited.

Similarly, VAS pain scores proved to be a critical component in differentiating the pain source. Rather than asking patients if a provocative maneuver like empty can testing hurts—evoking a binary yes-or-no answer—VAS pain scores allow patients to self-differentiate on a more granular scale. Again, our results show that most exam maneuvers evoke pain in the acutely painful shoulder. VAS scores provided granularity while adding minimal time. As seen in Codman for biceps vs. Codman for supraspinatus, supraspinatus produced the lowest mean VAS score in those that responded to CSI. It should be no surprise that Codman for supraspinatus in nonresponders to CSI had a nearly significantly higher mean VAS of 6.3 (*P* = .07), suggesting rotator cuff pathology as the pain generator rather than biceps tendonitis. By combining VAS score with the Codman maneuver, our examiners effectively differentiated biceps tendonitis from other shoulder pain generators.

Prior investigations of most shoulder maneuvers, including the Codman maneuver, focus on the ability of the exam to identify structural injuries to the rotator cuff, labrum, and long head of biceps. These injuries are then confirmed or refuted on MRI. Biceps tendonitis is not as reliably diagnosed on MRI, making evaluation of this Codman maneuver for biceps more difficult. Therefore, we chose ultrasound-guided CSI at the bicipital groove as both a diagnostic and therapeutic tool. This injection is relatively low cost and low risk.^16^^,^^20^ Rather than pursuing an MRI after an exam with global shoulder pains, our data supports the use of the Codman maneuver for biceps as justification for an ultrasound-guided CSI into the bicipital groove.

There are multiple limitations to this study. First, using response to an injection as a diagnostic tool limited the evaluation to those who were indicated for an injection, thus limiting the comparison group. Furthermore, injections have inherent confounding factors, such as technique and placebo effects. All injections were done by trained providers and utilized ultrasound to confirm location. Additionally, concomitant structural pathology likely exists in many of our patients. A positive finding of biceps pain that responds to injection may only be a part of the patient’s clinical picture. Continued pain, instability, or concerns on exam should warrant further evaluation with MRI. While MRI data on all participants could have enhanced our understanding of how this maneuver correlates with findings on imaging, many of our patients experienced significant relief with CSI, thus not pursuing further imaging. Finally, our study was powered for 28 participants but due to challenges with follow-up of a patient, 27 participants were included. We believe that the stark differences in VAS pain scores between patients in our cohort still provide clinically meaningful data.

## Conclusion

Our updated description of the Codman maneuver allows examiners to reliably find the long head of biceps tendon and the supraspinatus footprint. This exam identified patients who benefitted from ultrasound-guided CSI at the bicipital groove. Using VAS scores with the maneuver differentiated biceps tendonitis pain from supraspinatus tendinopathy better than traditional full and empty can maneuver.

## Disclaimers:

Funding: No funding was disclosed by the authors.

Conflicts of interest: Eric R. Wagner is a consultant for Stryker Corporation, Zimmer Biomet, Acumed, and Osteoremedies; he also receives institutional research support from Konica Minolta. Michael B. Gottschalk receives research support from Stryker Corporation, Konica Minolta, and Arthrex. The other authors, their immediate families, and any research foundation with which they are affiliated have not received any financial payments or other benefits from any commercial entity related to the subject of this article.

## References

[1] Arrigoni P., Ragone V., D'Ambrosi R., Denard P., Randelli F., Banfi G. (2014). Improving the accuracy of the preoperative diagnosis of long head of the biceps pathology: the biceps resisted flexion test. Joints.

[2] Codman E.A. (1934).

[3] Cotter E.J., Hannon C.P., Christian D., Frank R.M., Bach B.R. (2018). Comprehensive examination of the athlete’s shoulder. Sports Health.

[4] Gill T.K., Shanahan E.M., Allison D., Alcorn D., Hill C.L. (2014). Prevalence of abnormalities on shoulder MRI in symptomatic and asymptomatic older adults. Int J Rheum Dis.

[5] Gismervik S.O., Drogset J.O., Granviken F., Ro M., Leivseth G. (2017). Physical examination tests of the shoulder: a systematic review and meta-analysis of diagnostic test performance. BMC Musculoskelet Disord.

[6] Haefeli M., Elfering A. (2006). Pain assessment. Eur Spine J.

[7] Hegedus E.J., Goode A., Campbell S., Morin A., Tamaddoni M., Moorman C.T. (2008). Physical examination tests of the shoulder: a systematic review with meta-analysis of individual tests. Br J Sports Med.

[8] Hegedus E.J., Goode A.P., Cook C.E., Michener L., Myer C.A., Myer D.M. (2012). Which physical examination tests provide clinicians with the most value when examining the shoulder? Update of a systematic review with meta-analysis of individual tests. Br J Sports Med.

[9] Holtby R., Razmjou H. (2004). Accuracy of the Speed's and Yergason's tests in detecting biceps pathology and SLAP lesions: comparison with arthroscopic findings. Arthroscopy.

[10] Jain N.B., Luz J., Higgins L.D., Dong Y., Warner J.J., Matzkin E. (2017). The diagnostic accuracy of special tests for rotator cuff tear: the ROW cohort study. Am J Phys Med Rehabil.

[11] Jobe F.W., Moynes D.R. (1982). Delineation of diagnostic criteria and a rehabilitation program for rotator cuff injuries. Am J Sports Med.

[12] Kelly B.T., Kadrmas W.R., Speer K.P. (1996). The manual muscle examination for rotator cuff strength. An electromyographic investigation. Am J Sports Med.

[13] Leroux J.L., Thomas E., Bonnel F., Blotman F. (1995). Diagnostic value of clinical tests for shoulder impingement syndrome. Rev Rhum Engl Ed.

[14] van Moppes F.I., Veldkamp O., Roorda J. (1995). Role of shoulder ultrasonography in the evaluation of the painful shoulder. Eur J Radiol.

[15] O'Brien S.J., Pagnani M.J., Fealy S., McGlynn S.R., Wilson J.B. (1998). The active compression test: a new and effective test for diagnosing labral tears and acromioclavicular joint abnormality. Am J Sports Med.

[16] O'Kane J.W., Toresdahl B.G. (2014). The evidenced-based shoulder evaluation. Curr Sports Med Rep.

[17] Park H.B., Yokota A., Gill H.S., El Rassi G., McFarland E.G. (2005). Diagnostic accuracy of clinical tests for the different degrees of subacromial impingement syndrome. J Bone Joint Surg Am.

[18] Pogorzelski J., Fritz E.M., Godin J.A., Imhoff A.B., Millett P.J. (2018). Nonoperative treatment of five common shoulder injuries: a critical analysis. Obere Extrem.

[19] Ponce B.A., Kundukulam J.A., Sheppard E.D., Determann J.R., McGwin G., Narducci C.A. (2014). Rotator cuff crepitus: could Codman really feel a cuff tear?. J Shoulder Elbow Surg.

[20] Pourcho A.M., Colio S.W., Hall M.M. (2016). Ultrasound-guided interventional procedures about the shoulder: anatomy, indications, and techniques. Phys Med Rehabil Clin N Am.

[21] Rosas S., Krill M.K., Amoo-Achampong K., Kwon K., Nwachukwu B.U., McCormick F. (2017). A practical, evidence-based, comprehensive (PEC) physical examination for diagnosing pathology of the long head of the biceps. J Shoulder Elbow Surg.

[22] Winter S.B., Hawkins R.J. (2014). Comprehensive history and physical examination of the throwing shoulder. Sports Med Arthrosc Rev.

[23] Wolf E.M., Agrawal V. (2001). Transdeltoid palpation (the rent test) in the diagnosis of rotator cuff tears. J Shoulder Elbow Surg.

